# Sleep Quality Is Associated With Severe Meibomian Gland Disruption in Dry Eye

**DOI:** 10.3389/fmed.2022.812705

**Published:** 2022-02-17

**Authors:** Yirui Zhu, Xiaodan Huang, Lin Lin, Mengshu Di, Ruida Chen, Fei Fang, Xiuming Jin

**Affiliations:** ^1^Eye Center, Affiliated Second Hospital, School of Medicine, Zhejiang University, Hangzhou, China; ^2^Eye Department, Affiliated Dongyang Hospital, Wenzhou Medical University, Dongyang, China; ^3^Department of ENT, Wuning County People's Hospital, Jiujiang, China

**Keywords:** poor sleep quality, dry eye, MGD, meibomian gland dropout, Pittsburgh Sleep Quality Index (PSQI)

## Abstract

**Purpose:**

To investigate the association between sleep quality and meibomian gland dropout characteristics in dry eye patients.

**Methods:**

This cross-sectional study involved 172 dry eye patients with no history of conditions or factors that could confound dry eye disease (DED) and/or meibomian gland dropout. Participants underwent a comprehensive anterior eye assessment. The validated Athens Insomnia Scale (AIS) and Pittsburgh Sleep Quality Index (PSQI) were used to assess sleep quality. The measured outcomes were dry eye symptoms via the Ocular Surface Disease Index (OSDI), tear breakup time (TBUT), corneal fluorescein staining, meibomian gland function, and extent of meibomian gland dropout.

**Results:**

Of the dry eye participants, 34.9% had severe meibomian gland dropout (SMD) and 41.3% of the subjects had poor sleep quality. Patients with poor sleep quality had greater Meibomian gland dropout while the sleep AIS and PSQI scores were significantly correlated with Meibomian gland dropout (*r* = 0.495, *p* < 0.001; *r* = 0.24, *p* = 0.002; respectively). SMD patients had worse scores on all components of the PSQI (all *p* < 0.001, corrected for age and sex). Use of sleep medication, poor habitual sleep efficiency, and sleep disturbance were particularly prevalent in SMD patients as compared to Non-severe meibomian gland dropout (NSMD) patients. Multivariate logistic regression analysis revealed that sleep quality was eventually associated with female gender (*p* = 0.042), OSDI (*p* = 0.004), TBUT (*p* = 0.036), and Meibomian gland dropout score (*p* < 0.001).

**Conclusion:**

It was found that greater meibomian gland dropout in poor sleep quality individuals is especially related to use of sleep medication, poor habitual sleep efficiency, and sleep disturbance. This finding suggests a need for long-term studies of anterior eye health in people with poor sleep quality.

## Introduction

Sleep dysfunction leads to disruption of physiological systems essential to maintaining good health. Sleep disturbances, such as difficulties with sleep onset (sleep latency), efficiency, or duration, are not only highly prevalent in over one-third of the general population, with 8–27% reporting experiencing chronic or severe sleep problems, but are also associated with increased risk for serious diseases and health conditions, dependence on medication, higher incidences of drugs or alcohol abuse, and greater utilization of medical services (1, 2).

Dry eye disease (DED) is a multifactorial disease of the tears and ocular surface that results in symptoms of discomfort, visual disturbance, and tear film instability (3). The chronic discomfort observed in DED directly decreases quality of life and interferes with the ability to carry out daily functions (4–6). Meibomian gland dysfunction (MGD) is a leading cause of evaporative DED. MGD is characterized by terminal duct obstruction and altered meibum secretion. These obstructions block the delivery of meibum into the lid margin and cause an increase in pressure within the gland. The increased pressure results in atrophic gland degeneration, meibomian gland dropout, and ultimately results in the disappearance of glandular tissue within the upper and lower tarsal plates (7). Abnormal meibum quality and quantity can lead to a decreased tear film lipid layer, tear hyperosmolarity, mechanical irritation through increased friction, and the onset of inflammatory cascades, all of which can lead to ocular surface damage (7–9). Meibomian gland dropout and altered quality of expressed meibum have been used to classify the severity of MGD (8).

DED is commonly observed in individuals with sleep disturbances and other related disorders. A large, community-based study conducted by our eye center showed a strong association between poor sleep quality and an increased severity of dry eye (10). Mengliang et al. (11) found that sleep quality plays an important role in the development of dry eye by influencing tear secretion and tear film stability by indirectly aggravating anxiety and depression. Furthermore, sleep deprivation in a mouse model study indicated that sleep deprivation induced dry eye by disrupting superficial corneal epithelial cells microvilli morphology, which is caused by sequential declines in the peroxisome proliferator-activated receptor alpha (PPARα), transient receptor potential vanilloid 6 (TRPV6) expression, and Ezrin phosphorylation status (12). Though these studies suggest that sleep deprivation may contribute to dry eye status, they have not identified differences in meibomian gland structure in subjects with and without sleep deprivation in dry eye patients. Additionally, few studies to date have further analyzed the relationship between meibomian glands architecture parameters and poor sleep quality.

This study had several objectives. First, the purpose of the present study was to compare meibomian gland dropout and meibum quality between dry eye patients with and without sleep deprivation. Second, we planned to determine the association between sleep quality and meibum grade scores. Finally, we sought to assess the strength of the association between sleep quality and meibomian gland dropout excluding other chronic conditions known to induce meibomian gland loss. Our findings explored increasing severity of meibomian gland dropout in dry eye patients with the likelihood of having poor sleep quality.

## Patients and Methods

### Patients

This study was approved by the Institutional Review Board of the Affiliated Second Hospital of Zhejiang University. The research followed the tenets of the Declaration of Helsinki. In this cross-sectional study, patients with DED were recruited at the eye clinic of the Affiliated Second Hospital of Zhejiang University (Hangzhou, China) and underwent standardized assessments from June 1, 2020 to May 30, 2021 (*N* = 172). DED was diagnosed in accordance with the following characteristics: 1) dry eye symptoms; 2) positive corneal fluorescein staining; and 3) a Schirmer I test result <5 mm or a tear breakup time (TBUT) <5 s.

Patients were excluded from the study if they presented with conditions or factors that could confound DED and meibomian gland dropout including eyelid or ocular surface disorders, allergic conjunctivitis, glaucoma, macular degeneration, contact lens-wear, any active external ocular processes, eye tattoo history, or a history of ocular surgery. Subjects with systemic conditions knowingly associated with ocular surface disease were also excluded from the study. These included anxiety, depression, diabetes mellitus, autoimmune disease, thyroid disease, vitamin B12 deficiency, oral isotretinoin therapy, patients taking oral hormone level drugs, and patients diagnosed with obstructive sleep apnea.

Tear meniscus height (TMH) and meibography were performed by capturing infrared images with the noncontact meibography system Oculus Keratograph 5 M (Oculus, Wetzlar, Germany). The meibomian gland dropout was defined as the percentage of gland dropout in relation to the total tarsal area of the upper and lower eyelids. The patients were classified into two groups in accordance with the severity of meibomian gland dropout: (1) Non-severe meibomian gland dropout group (NSMD group; where the affected area was <1/2 of the total area occupied by meibomian glands); (2) severe meibomian gland dropout group (SMD group; where the affected area was >2/3 of the total area occupied by meibomian glands). The demographic characteristics of the patients in each group are shown in Table 1. For each individual, demographic information (age, sex, residence, level of education, working status, financial status) were collected.

**Table 1:** Demographic characteristics of study subjects: sleep characteristics and dry eye parameters.

	**MG evaluation**			**Sleep evaluation**		
**Parameters**	**Group with NSMD**	**Group with SMD**	***p*-value**	**Group with good sleep**	**Group with poor sleep**	***p*-value**
Number of subjects	112 (65.1%)	60 (34.9%)	–	101 (58.7%)	71 (41.3%)	–
Age, years	46.3 ± 11.1	51.0 ± 8.8	0.003^*^	46.2 ± 11.2	50.4 ± 9.1	0.003^*^
Gender			0.227			0.007^*^
Male	40 (35.7%)	16 (26.7%)	–	41 (40.6%)	15 (21.1%)	–
Female	72 (64.3%)	44 (73.3%)	–	60 (59.4%)	56 (78.9%)	–
Residence			0.506			0.13
Rural	67 (59.8%)	39 (65%)	–	67 (66.3%)	39 (54.9%)	–
Urban	45 (40.2%)	21 (35%)	–	34 (33.7%)	32 (45.1%)	–
Level of education			0.681			0.562
Illiteracy	9 (8.0%)	4 (6.7%)	–	8 (7.9%)	5 (7.1%)	–
Primary school	13 (11.6%)	11 (18.3%)	–	13 (12.8%)	11 (15.5%)	–
Middle school	38 (33.9%)	19 (31.7%)	–	30 (29.7%)	27 (38%)	–
College or more	52 (46.5%)	26 (43.3%)	–	50 (49.6%)	28 (39.4%)	–
Working status			0.961			0.233
Employed	78 (69.6%)	42 (70%)	–	74 (73.3%)	46 (64.8%)	–
Unemployed	34 (30.4%)	18 (30%)	–	27 (26.7%)	25 (35.2%)	–
Financial status			0.733			0.701
Low	21 (18.7%)	11 (18.3%)	–	17 (16.8%)	13 (18.3%)	–
Medium	63 (56.2%)	37 (61.7%)	–	57 (56.4%)	43 (60.6%)	–
High	28 (25.1%)	12 (20%)	–	27 (26.8%)	15 (21.1%)	–
OSDI	39.9 ± 22.3	53.2 ± 17.9	<0.001^*^	39.6 ± 21.4	51.7 ± 20.4	<0.001^*^
TBUT, s	5.2 ± 2.3	3.6 ± 1.8	<0.001^*^	5.1 ± 2.3	3.9 ± 2.1	<0.001^*^
Corneal staining	0.2 ± 0.5	0.4 ± 0.7	0.012^*^	0.3 ± 0.6	0.3 ± 0.7	0.738
TMH	0.17 ± 0.05	0.16 ± 0.05	0.343	0.17 ± 0.05	0.17 ± 0.07	0.351
MG quality	1.5 ± 0.9	2.2 ± 0.9	<0.001^*^	1.6 ± 0.9	1.9 ± 0.9	0.031^*^
MG expressibility	2.0 ± 0.8	2.9 ± 0.9	<0.001^*^	2.1 ± 0.9	2.6 ± 0.9	0.001^*^
MG dropout score	1.7 ± 1.8	9.9 ± 1.7	<0.001^*^	3.0 ± 3.6	6.9 ± 4.3	<0.001^*^
Sleep AIS score	3.5 ± 3.7	8.8 ± 4.9	<0.001^*^	2.0 ± 1.7	10.3 ± 3.6	<0.001^*^
Sleep PSQI score	3.7 ± 4.3	11.4 ± 6.9	<0.001^*^	2.0 ± 2.3	12.7 ± 5.3	<0.001^*^

* *p-values < 0.05 were considered significant*. *NSMD, Non-severe meibomian gland dropout; SMD, severe meibomian gland dropout; MG, meibomian gland; TBUT, tear film break-up time; OSDI, ocular surface disease index; TMH, tear meniscus height*.

### Evaluation of Dry Eye Symptoms

Before any examination, all patients completed the Ocular Surface Disease Index (OSDI) questionnaire to assess the severity of ocular surface symptoms. The OSDI questionnaire consists of 12 questions regarding the presence and frequency of symptoms related to the ocular surface (13).

The OSDI total score (ranging from 0 to 100) can be calculated with a formula using the sum score of all completed questions. The final scale ranges from 0 to 12 (no disability), 13 to 22 (mild symptoms), 23 to 32 (moderate symptoms), and 33 to 100 (severe symptoms).

### Sleep Quality Assessment

The validated Athens Insomnia Scale (AIS) and Pittsburgh Sleep Quality Index (PSQI) were used to assess sleep quality. AIS is an eight-item self-assessment psychometric instrument designed for quantifying sleep difficulty. For each item, four answers are provided representing increasing difficulty with sleep. Among these options are no problem, minor problem, considerable problem, and serious problem or did not sleep at all. Answers are scored 0, 1, 2, and 3, respectively. The total score ranges from 0 to 24, with greater scores depicting poorer sleep quality. A total score of 6 or higher identifies 90% of the subjects suffering from nonorganic insomnia (14).

The PSQI is the most frequently used validated questionnaire for assessing sleep quality (15). This self-reported questionnaire of Chinese version assesses the average sleep quality over the patient's last month of sleep. It consists of 19 questions in seven domains: subjective sleep quality, sleep latency, sleep duration, habitual sleep efficiency, sleep disturbances, use of sleep medication, and daytime dysfunction. The scores in each of these seven domains, each on a 0–3 scale, are summed to generate a global score with a possible range of 0–21. A global score of >5 was used as the cut-off to distinguish good sleepers (≤ 5) from poor sleepers (>5) (15).

### Evaluation of Dry Eye Signs

Dry eye tests were performed in both eyes in the following order: TBUT, corneal fluorescein staining, and MGD evaluation. As previously reported, corneal fluorescein staining was measured using commercially available sterile fluorescein paper strips (Jinming New Technological Development Co. Ltd., Tianjin, China) (16). The strip was moistened with 20 μL 0.9% sterile saline and gently pressed along the lower tarsal conjunctiva. The TBUT was then measured by counting the seconds after a blink before the tear film was broken. A median of three measurements per eye was taken. The TBUT ranged from 0 to 10. Corneal fluorescein staining score was assessed by grading the upper, middle, and lower parts of the cornea on a nine-point scale: no staining = 0; <5 stained punctate dots = 1; 5 to 9 stained punctate dots = 2; and ≥ 10 stained punctate dots or filamentous staining = 3. Afterwards, the total score for corneal fluorescein staining was calculated as the summed score for all three parts of the cornea and ranged from 0 to 9 (17). Meibomian gland function was evaluated in accordance with the recommendations of the International Workshop on Meibomian Gland Dysfunction (18, 19). MGD was assessed by averaging the quality score (clear = 1; cloudy = 2; granular = 3; toothpaste = 4) and expressibility score of the meibum (minimal pressure = 0; light pressure = 1; moderate pressure = 2; heavy pressure = 3).

### Evaluation of Meibomian Gland Morphology

**Figure 1:**
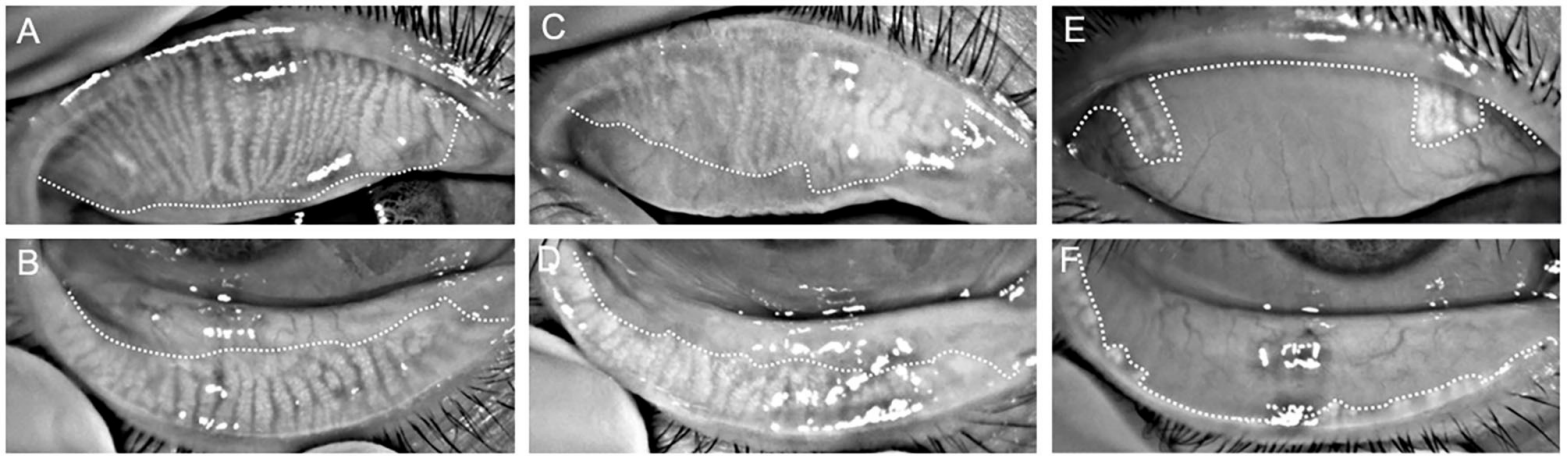
Representative infrared meibography images from the superior **(A,C,E)** and inferior **(B,D,F)** eyelids of a control **(A–D)** and poor sleep **(E,F)** participant. Overall, the poor sleep quality participant demonstrates more extensive MG dropout than the control participant.

As shown in Figure 1, partial or complete dropout of meibomian glands was scored using the following meiboscore grades: 0 = no loss; 1 = loss of an area of <1/3 of the total area; 2 = loss of an area between one-third and two-thirds of the total area; 3 = loss of an area of more than two-thirds of the total area. The meiboscores for the upper and lower eyelids were summed for each eye (20), and were evaluated by one examiner. Images were digitally analyzed using ImageJ software (National Institutes of Health). Photographs were analyzed in a random order by two double-blinded investigators to minimize observer bias.

### Statistical Analysis

Data analysis was performed using SPSS statistical software (SPSS Inc., Chicago, IL, USA). Continuous variables were expressed as mean ± SD and categorical variables were expressed as absolute and relative (%) frequencies. The data of only the right eye from all subjects were used for analysis. The Mann-Whitney *U* test was used to compare the ocular surface changes. Categorical variables were compared with the χ^2^ test. Correlations (Pearson and Spearman) were used to evaluate the strength of association between sleep and severity of meibomian gland dropout signs and symptoms. Logistic regression models were used to assess the relationship between poor sleep quality (PSQI score > 5 as the dependent variable) and meibomian gland dropout (meibomian gland dropout ≥2 as the independent variables). To investigate which components of sleep quality were most affected, we performed the same analysis for the seven components of the PSQI separately. The analysis was stratified by age in decades and sex. Multivariate logistic regression analysis was used to investigate the effects of various parameters on meibomian dropout. ORs and 95% CIs were presented. A *p*-value of < 0.05 was considered statistically significant.

## Results

During the study period between June 1, 2020 and May 30, 2021, an initial dataset was collected on a total of 286 patients with dry eye that had an ocular surface evaluation and sleep quality assessment. After the filtering process, 172 patients were included.

Out of the 172 dry eye participants, 60 (34.9%) subjects had severe meibomian gland dropout and 71 (41.3%) subjects had poor sleep quality. The subjects in NSMD and SMD groups did not differ significantly in terms of gender (*p* = 0.227), residence (*p* = 0.506), level of education (*p* = 0.681), working status (*p* = 0.961), and gross income (*p* = 0.733). The mean age of SMD participants was 51.0 ± 8.8 years (range: 26–75) and 44 were women (73.3%). Patients with SMD were older (*p* = 0.003), experienced more dry eye symptoms (*p* < 0.001), had worse meibomian gland function (*p* < 0.001), and had poorer sleep quality (*p* < 0.001) as compared to patients without SMD (Table 1). Patients with poor sleep were generally older (*p* = 0.003), were more frequently women (*p* = 0.007), experienced more dry eye symptoms (*p* < 0.001), and had greater meibomian gland dropout (*p* < 0.001) as compared to patients without poor sleep (Table 1).

The mean sleep AIS score was significantly higher in subjects with SMD than in subjects with NSMD (8.8 ± 4.9 vs. 3.5 ± 3.7, *p* < 0.001). Similarly, patients with poor sleep quality had greater Meibomian gland dropout than patients without poor sleep (6.9 ± 4.3 vs. 3.0 ± 3.6, *p* < 0.001) and the sleep AIS score and PSQI score were significantly correlated to Meibomian gland dropout (*r* = 0.495, *p* < 0.001; *r* = 0.24, *p* = 0.002; respectively; Table 2). Comparably, Meibomian gland dropout score was significantly associated with Meibomian gland quality (*r* = 0.303; *p* < 0.001), Meibomian gland expressibility (*r* = 0.478; *p* < 0.001), dry eye symptoms (*r* = 0.34; *p* < 0.001), TBUT (*r* = −0.691; *p* < 0.001^*^), and corneal staining (*r* = 0.751; *p* < 0.001). Nevertheless, the sleep PSQI score was not significantly associated with corneal staining (*r* = 0.079; *p* = 0.3) or Meibomian gland quality (*r* = 0.084; *p* = 0.271).

**Table 2:** Correlation between sleep quality and dry eye parameters.

**Variables**	**OSDI**	**TBUT**	**Corneal staining**	**MG quality**	**MG expressibility**	**MG dropout**
TBUT, s	*r* = −0.284					
	*p* < 0.001^*^					
Corneal staining	*r* = 0.245	*r* = −0.627				
	*p* = 0.001^*^	*p* < 0.001^*^				
MG quality	*r* = 0.062	*r* = −0.359	*r* = 0.246			
	*p* = 0.418	*p* < 0.001^*^	*p* = 0.001^*^			
MG expressibility	*r* = 0.254	*r* = −0.454	*r* = 0.343	*r* = 0.551		
	*p* = 0.001^*^	*p* < 0.001^*^	*p* < 0.001^*^	*p* < 0.001^*^		
MG dropout	*r* = 0.34	*r* = −0.691	*r* = 0.751	*r* = 0.303	*r* = 0.478	
	*p* < 0.001^*^	*p* < 0.001^*^	*p* < 0.001^*^	*p* < 0.001^*^	*p* < 0.001^*^	
Sleep AIS score	*r* = 0.248	*r* = −0.434	*r* = 0.318	*r* = 0.2	*r* = 0.238	*r* = 0.495
	*p* = 0.001^*^	*p* < 0.001^*^	*p* < 0.001^*^	*p* = 0.008^*^	*p* = 0.002^*^	*p* < 0.001^*^
Sleep PSQI score	*r* = 0.292	*r* = −0.189	*r* = 0.079	*r* = 0.084	*r* = 0.17	*r* = 0.24
	*p* < 0.001^*^	*p* = 0.013^*^	*p* = 0.3	*p* = 0.271	*p* = 0.026^*^	*p* = 0.002^*^

* *p-values < 0.05 were considered significant*. *OSDI, ocular surface disease index; MG, meibomian gland; TBUT, tear film break-up time; PSQI, pittsburgh sleep quality index*.

Table 3 presents the relationship between sleep quality and Meibomian gland dropout. In SMD patients, poor sleep quality was much more prevalent than in NSMD groups (3.7 ± 4.3 vs. 11.4 ± 6.9; *p* < 0.001; OR 1.21 [95% CI 1.13–1.29], corrected for age and sex only, *p* < 0.001; OR 1.21 [95% CI 1.13–1.30], corrected for age, sex, education, residence, income and working status, *p* < 0.001). Table 3 further demonstrates the association between Meibomian gland dropout and different components of sleep quality. It reveals that SMD patients have worse scores than NSMD patients on all components of the PSQI (all *p* < 0.001, corrected for age and sex). Use of sleep medication, poor habitual sleep efficiency, and sleep disturbance were particularly prevalent in SMD patients as compared to NSMD patients. After correcting for age, sex, education, residence, income, and working status, SMD was still associated with lower sleep quality on all seven components. The multivariate logistic regression analysis included presence or absence of poor sleep as dependent parameters, with demographics and dry eye variables as independent parameters by dropping those parameters with a *p* value > 0.05. Sleep quality was eventually associated with female gender (*p* = 0.042) and poorer scoring on OSDI (*p* = 0.004), TBUT (*p* = 0.036), and Meibomian gland dropout scores (*p* < 0.001) (Table 4).

**Table 3:** Association between sleep quality (all components of the PSQI) and MG dropout.

**PSQI component**	**Without SMD**	**With SMD**	***p* value**	**Corrected for age and sex only**		**Corrected for age, sex, education, residence, income, working status**	
				**OR (95% CI)**	***p*-value**	**OR (95% CI)**	***p*-value**
Global score: Overall poor quality of sleep (PSQI > 5)	3.7 ± 4.3	11.4 ± 6.9	<0.001^*^	1.226 (1.145–1.312)	<0.001^*^	1.248 (1.158–1.346)	<0.001^*^
1. Subjective sleep quality (bad vs. good)	1.2 ± 1.1	2.3 ± 0.8	<0.001^*^	2.428 (1.696–3.476)	<0.001^*^	2.614 (1.773–3.853)	<0.001^*^
2. Suboptimal sleep latency (>2 vs. ≤ 2)	0.5 ± 0.8	1.4 ± 1.2	<0.001^*^	2.023 (1.439–2.843)	<0.001^*^	2.182 (1.521–3.132)	<0.001^*^
3. Suboptimal sleep duration (>6 h vs. ≤ 6 h)	0.6 ± 0.8	1.7 ± 1.2	<0.001^*^	2.902 (1.991–4.231)	<0.001^*^	3.205 (2.118–4.849)	<0.001^*^
4. Poor habitual sleep efficiency (<75% vs. ≥75%)	0.4 ± 0.6	1.6 ± 1.2	<0.001^*^	3.798 (2.464–5.857)	<0.001^*^	4.226 (2.631–6.787)	<0.001^*^
5. Sleep disturbance (>9 vs. ≤ 9)	0.5 ± 0.8	1.9 ± 1.1	<0.001^*^	3.439 (2.338–5.057)	<0.001^*^	4.495 (2.766–7.305)	<0.001^*^
6. Use of sleep medication (yes vs. no)	0.2 ± 0.4	1.1 ± 1.1	<0.001^*^	4.37 (2.532–7.541)	<0.001^*^	4.554 (2.581–8.037)	<0.001^*^
7. Daytime dysfunction (>2 vs. ≤ 2)	0.4 ± 0.7	1.4 ± 1.1	<0.001^*^	2.846 (1.911–4.24)	<0.001^*^	3.415 (2.152–5.421)	<0.001^*^

* *p-values < 0.05 were considered significant*. *PSQI, pittsburgh sleep quality index; MG, meibomian gland; SMD, severe meibomian gland dropout; CI, confidence interval; OR, odds ratio*.

**Table 4:** Multivariate logistic regression analysis between sleep quality (PSQI score), demographics and dry eye parameters.

**Parameters**	**OR**	***p* value**	**95% CI**	
			**Lower**	**Upper**
Age	0.001	*p* = 0.991	−0.118	0.119
Female gender	0.129	*p* = 0.042^*^	0.068	3.519
Rural residence	0.044	*p* = 0.496	−1.118	2.296
Level of education	0.007	*p* = 0.935	−1.136	1.233
Gross income	0.032	*p* = 0.684	−1.256	1.909
Working status	0.122	*p* = 0.154	−0.658	4.120
OSDI	0.195	*p* = 0.004^*^	0.018	0.098
TBUT, s	−0.145	*p* = 0.036^*^	−0.790	−0.028
Corneal staining	−0.046	*p* = 0.476	−1.770	0.829
TMH	−0.037	*p* = 0.572	−18.437	10.213
MG quality	−0.014	*p* = 0.857	−1.140	0.950
MG expressibility	−0.047	*p* = 0.580	−1.424	0.800
MG dropout score	0.467	*p* < 0.001^*^	0.481	0.928

* *p-values < 0.05 were considered significant*. *PSQI, pittsburgh sleep quality index; MG, meibomian gland; TBUT, tear film break-up time; OSDI, ocular surface disease index; TMH, tear meniscus height; CI, confidence interval; OR, odds ratio*.

## Discussion

Although previous studies have conducted meibomian gland evaluation and quantification in individuals with poor sleep, during this study we undertook a novel and comprehensive approach in characterizing anterior ocular health and dry eye disease in people with and without poor sleep (11). In doing so, the major finding of this cross-sectional study revealed the presence of more extensive meibomian gland dropout in poor sleep quality individuals relative to that of the control participants.

It was determined that poor sleepers with worse outcomes in all components of the PSQI were more likely to have severe meibomian gland dropout. Moreover, patients with dry eye with poor sleep had shorter TBUT and higher meibum grade scores that were in accordance with previous studies that showed a positive association between dry eye and sleep quality (11). The result of severe meibomian gland dropout in participants with reduced quality of sleep may emphasize the clinical value of this association and help increase awareness of sleep disease as a serious disorder affecting multiple aspects of ocular surface health.

Meibomian glands provide key components to the tear film that help maintain a healthy ocular surface. When these glands are reduced, absent, or dysfunctional, the impact on the ocular surface can be immense (21). Clinical studies have reported that patients with obstructive MGD (defined as meibomian gland dropout, poor meibum expression, and lack of signs of inflammation) had increased tear evaporation rates compared with controls (22). If alterations in meibum lipid composition occur in MGD pathogenesis, meibum cannot function normally and can increase tear film evaporation, which results in evaporative dry eyes and corneal staining (23). Our study supports this understanding of pathogenesis based on the correlations between expressed meibum grade, TBUT, and corneal staining.

Meibomian gland dropout is typically caused by atrophic degeneration secondary to increased pressure within the gland because of orifice obstruction and meibomian gland duct obliteration in the late phase of the disease (8, 24). In a cross-sectional study, Bao et al., reported the possible mechanism of an immune-driven process underlying meibomian gland dropout in early HIV (25). Another prospective cross-sectional study found that meibomian gland dropout is related to indoor microbial concentration (26). As found in other studies, aging is also a known risk factor for MGD (27). With age, meibomian glands exhibit decreased meibocyte differentiation, decreased meibomian gland size, increased meibomian gland dropout, and increased inflammatory cell infiltration (28). Additionally, age is a significant factor when evaluating the clinical signs of dry eye and sleep quality (29, 30). Consistent with previous studies, we found that most older individuals showed more glandular loss and had poorer sleep. Therefore, we analyzed the relationship between meibomian gland dropout score and sleep quality after correcting for age.

Past studies investigating the association between poor sleep quality and dry eye have shown that the relationship is likely to be complex. Several studies have gone so far as to correlate DED not only with sleep quality, but sleep position (31, 32). Mengliang et al. reported that sleep quality plays an important role in the development of DED in influencing tear secretion and tear film stability by indirectly aggravating anxiety and depression, ultimately leading to higher self-reported symptom scores (11). Similarly, a large population-based study found that dry eye is associated with worse outcomes in all quantitative and qualitative aspects of sleep, and this relationship is present in all ages and sexes (33). An randomized control trial showed that the repeated use of warming eye masks had a positive effect on both tear function and mental health including depression and anxiety (34). An recent study suggested that the inability to close the eyelids during sleep was associated with worsened DED symptoms and poor sleep quality (35). The results might provide additional evidence for a clinical association between DED and sleep. This relationship between sleep quality and dry eye is partly explained by coexisting comorbidities such as autoimmune diseases, psychiatric disorders, endocrine disorders, and chronic pain syndromes. Therefore, patients were excluded from the study if they presented with conditions or factors that could confound DED and meibomian gland dropout. Within this current study, results showed noticeable changes in tear film break-up time and meibomian gland function in sleep deprivation subjects. In previous studies, sleep deprivation induced dry eye through the defective changes of superficial corneal epithelial cells in a mouse model (12). This study also revealed that meibomian gland morphology after sleep deprivation for 5–10 days was the same as that of normal mice. One possibility for explanation is that short-term sleep deprivation could not cause meibomian gland morphology change. Considering all of this, it is clear that all the mechanisms underlying the association between sleep quality and meibomian gland function are still not fully understood. Further studies in understanding the underlying biological mechanisms linked to sleep deprivation disorder are warranted.

In the present study it was demonstrated that SMD is clearly associated with various sleep parameters, particularly use of sleep medication, poor habitual sleep efficiency, and sleep disturbance. This highlights the impact of sleep quality on a patient's life, but also the need for holistic thinking when treating a patient with DED. It is important to address comorbid conditions such as systemic conditions, poor sleep, depression, and anxiety. As these conditions are thought to exacerbate one another and contribute to the overall burden of disease, treatment options should be considered.

Our study has several limitations. First, as this was a cross-sectional study, we were unable to infer causality on any of the reported associations. Longitudinal studies with a larger sample size are required to further clarify these relationships. Second, since some study participants could not answer the sleep quality questionnaire alone, these questions were asked by trained technicians for all subjects. Other limitations of this study also subject to recall bias of self-reported sleep parameters. Nevertheless, self-reported assessments of sleep have been shown to be valid measures compared with quantitative sleep assessments with actigraphy (36). Moreover, the effects of risk factors, such as menopause, body mass index, social status, and other possible confounding factors, should be considered to ensure a detailed understanding of the relationships among sleep status and SMD. In addition, we excluded individuals with a known diagnosis of autoimmune diseases, psychiatric disorders, endocrine disorders, and chronic pain syndromes, but did not conduct laboratory screening for this potential confounder in the present study.

In conclusion, poor sleep quality is significantly associated with severe meibomian gland dropout. These findings suggest that clinicians should be aware of this relationship and ask about dry eye symptoms and sleep quality, especially in highly symptomatic patients. Further prospective studies are needed to determine the directionality of this association and establish appropriate treatment strategies for patients with poor sleep quality and severe meibomian gland dropout.

## Data Availability Statement

The original contributions presented in the study are included in the article/supplementary material, further inquiries can be directed to the corresponding author.

## Ethics Statement

The studies involving human participants were reviewed and approved by Institutional Review Board of the Affiliated Second Hospital of Zhejiang University. The patients/participants provided their written informed consent to participate in this study.

## Author Contributions

All authors listed have made a substantial, direct, and intellectual contribution to the work and approved it for publication.

## Conflict of Interest

The authors declare that the research was conducted in the absence of any commercial or financial relationships that could be construed as a potential conflict of interest.

## Publisher's Note

All claims expressed in this article are solely those of the authors and do not necessarily represent those of their affiliated organizations, or those of the publisher, the editors and the reviewers. Any product that may be evaluated in this article, or claim that may be made by its manufacturer, is not guaranteed or endorsed by the publisher.
